# Predictors of health-related quality of life in outpatients with coronary heart disease

**DOI:** 10.3389/fpsyg.2023.1119093

**Published:** 2023-06-09

**Authors:** Lars Aastebøl Frøjd, John Munkhaugen, Costas Papageorgiou, Elise Sverre, Torbjørn Moum, Toril Dammen

**Affiliations:** ^1^Department of Medicine, Drammen Hospital, Drammen, Norway; ^2^Institute of Clinical Medicine, Faculty of Medicine, University of Oslo, Oslo, Norway; ^3^Department of Behavioural Medicine, University of Oslo, Oslo, Norway; ^4^Asto Clinics, Cheshire, United Kingdom; ^5^Institute of Psychology, University of Oslo, Oslo, Norway; ^6^Department of Mental Health and Addiction, Oslo University Hospital, Oslo, Norway

**Keywords:** coronary heart disease, type D personality, depression, insomnia, secondary prevention, anxiety, quality of life, sleep initiation and maintenance disorders

## Abstract

**Introduction:**

Health-related quality of life (HRQoL) is an important treatment target in patients with coronary heart disease (CHD) and is associated with poor outcomes. Therefore, it is of clinical importance to identify the key determinants of HRQoL among these patients. There is, however, limited knowledge of how a comprehensive set of psychosocial factors influence HRQoL. We aimed to determine the relative associations of clinical and psychosocial factors with mental and physical components of HRQoL in a sample of CHD outpatients.

**Methods:**

This cross-sectional study included 1,042 patients 2–36 (mean 16) months after a CHD event recruited from two general Norwegian hospitals with a combined catchment area making up 7% of the Norwegian population, representative with regards to demographic and clinical factors. We collected data on HRQoL, demographics, comorbidities, coronary risk factors, and psychosocial factors. HRQoL was assessed using the Short Form 12 (SF12), which comprises a Mental Component Scale (MCS), and the Physical Component Scale (PCS). Crude and multi-adjusted linear regression analyses were used to investigate the association between covariates and MCS and PCS.

**Results:**

Mean age was 61 [standard deviation (SD) 10] years, 20% were females, 18% had type D personality, 20% significant depression symptoms, 14% significant symptoms of anxiety whereas 45% reported insomnia. The presence of type D personality (β: −0.19), significant symptoms of depression (β: −0.15), and the presence of insomnia (β: −0.13) were negatively associated with MCS, but not PCS in multi-adjusted analyses. The presence of chronic kidney disease (β: −0.11) was associated with reduced MCS, whereas the presence of chronic obstructive pulmonary disease (β: −0.08) and low physical activity (β: −0.14) were negatively associated with PCS. Younger age was associated with lower MCS, whereas older age was associated with lower PCS.

**Discussion:**

We conclude that Type D personality, depressive symptoms, insomnia, and chronic kidney disease were the strongest determinants of the mental component of HRQoL. Assessing and managing these psychological factors among CHD outpatients may improve their mental HRQoL.

## 1. Introduction

The increased survival rates after coronary heart disease (CHD) events, such as myocardial infarction, and an aging population result in more patients living with chronic coronary syndrome (Mensah et al., 2017). CHD remains a leading cause of Disability Adjusted Life Years worldwide (Roth et al., 2020). This has led to an increased need for effective secondary prevention. Living with CHD may lead to a life with disability, which is closely related to reductions in health-related quality of life (HRQoL) (Xie et al., 2008; Mols et al., 2009; Palacios et al., 2016). HRQoL is a generic assessment of mental and physical health and is often used to estimate the burden of diseases and treatments (Ware and Gandek, 1998). Whereas interventional studies and studies of prognostic factors in CHD patients have mainly focused on end-points in terms of major adverse cardiac events (Visseren et al., 2021), there is limited knowledge about how a comprehensive set of factors affect HRQoL cross-sectionally in CHD outpatients (Palacios et al., 2018).

Sociodemographic and clinical factors such as low education (Muhammad et al., 2014), somatic comorbidities (i.e., chronic heart failure, transient ischemic attack) (McBurney et al., 2002), and low drug adherence (McBurney et al., 2002) have been associated with poor HRQoL as assessed with the Short- Form 12 (SF12) HRQoL questionnaire in CHD patients. On the other hand, studies on the association between both sociodemographic, clinical, and a comprehensive set of psychosocial factors and HRQoL are sparse. Only a limited number of relevant factors have been studied so far with assessments made within the first year after the index event (McBurney et al., 2002; Palacios et al., 2018; Christensen et al., 2020; Mayer et al., 2020). Increasing symptoms of anxiety and depression have been associated with both lower scores on the MCS subscale and the PCS subscale of SF12 (Muhammad et al., 2014; Palacios et al., 2016) and total score of Short-Form 36 (SF36) in CHD patients (Mayer et al., 2020). Furthermore, Type D (“distressed”) personality has been associated with reduced cardiovascular disease-specific quality of life in CHD patients (Jo et al., 2019). Type D personality is defined as a combination of the personality traits Negative Affectivity (NA) and Social Inhibition (SI) (Denollet, 2005) and has been associated with cardiovascular risk factors as well as with negative prognosis (Denollet, 2000; Kupper and Denollet, 2018). NA is a propensity to experience negative emotions, while SI is a tendency to inhibit self-expression in social situations (Denollet, 2005).

More recently, insomnia has also been identified as being highly prevalent in CHD patients with estimates ranging from 36% to 45% (Coryell et al., 2013; Da Costa et al., 2017; Frøjd et al., 2021). We have also identified insomnia as a key factor for poor prognosis in CHD outpatients (Frøjd et al., 2022). Insomnia is characterized by difficulties with sleep initiation, maintenance, or non-restorative sleep, coupled with daytime impairment (American Psychiatric Association, 2000). There are some studies indicating an association between insomnia symptoms and reductions in HRQoL in the general population (Tang et al., 2017; Franquelo-Morales et al., 2018; Matsui et al., 2021). However, to the best of our knowledge, no previous studies have investigated the impact of insomnia on mental and physical components of HRQoL in CHD patients.

Importantly, no previous studies have focused on the relative importance of insomnia, Type-D personality, anxiety, and depression on the mental and physical components of HRQOL. These psychosocial factors overlap and are multidirectionally related (De Smedt et al., 2014; Le Grande et al., 2016; Javaheri and Redline, 2017). Reduced HRQoL is an important target for treatment and is associated with adverse clinical outcomes (Westin et al., 2005). There is a need to know the relative importance on HRQoL to better tailor future interventional studies targeting the most important factors important to HRQoL. Therefore, we aimed to determine the relative importance of these factors on mental and physical HRQoL in CHD outpatients. We hypothesized that potentially modifiable psychosocial variables (e.g., anxiety, depression, and insomnia) as well as lower age would be key determinants independently associated with reduced MCS in CHD outpatients. Moreover, we hypothesized that depression, as well as increasing age, low education, and, somatic comorbidities (e.g., congestive heart failure and transient ischemic attack) would be associated with reduced PCS in this population.

## 2. Materials and methods

### 2.1. Study design and population

**FIGURE 1:**
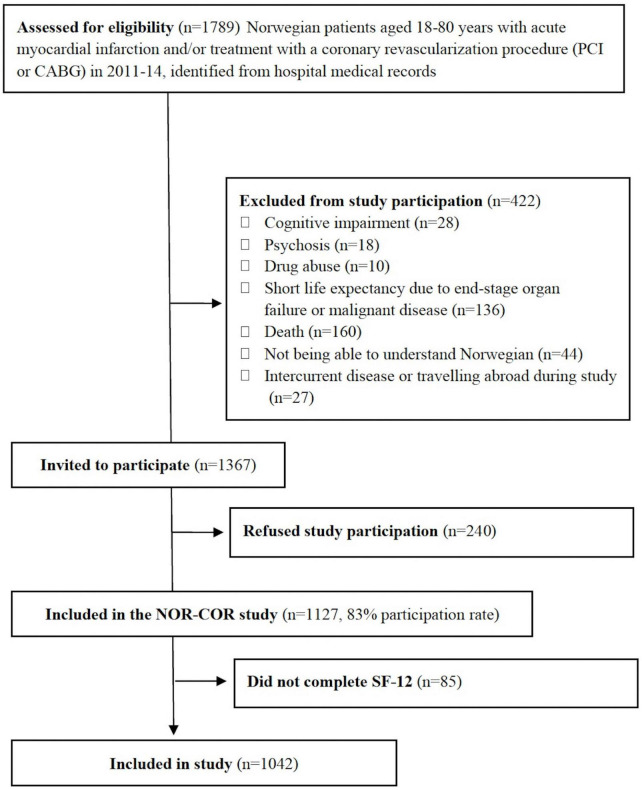
Study flow chart. CABG, coronary artery bypass grafting; PCI, percutaneous coronary intervention; SF-12, Short form 12.

The NORwegian CORonary prevention study (NOR-COR) is a cross-sectional study. An in-depth description of methods and baseline characteristics has been published elsewhere (Munkhaugen et al., 2016). Patients aged 18–80 years with a first or recurrent coronary event, defined as acute myocardial infarction and/or a revascularization procedure coronary artery bypass grafting or percutaneous coronary intervention, were evaluated for eligibility, based on hospital discharge lists between 2011 and 2014. Patients were recruited from two general Norwegian hospitals (Drammen and Vestfold) with a combined catchment area of 380,000 inhabitants, making up 7% of the Norwegian population. The catchment area is representative of the Norwegian population with regards to demographic and clinical factors (Munkhaugen et al., 2016). Some patients had several coronary events, and the index event was defined as the last event recorded prior to the time of study inclusion. Briefly, 1,789 patients (18–80 years) with acute myocardial infarction and/or a revascularization procedure were evaluated for study participation. Flow chart with inclusion and exclusion criteria is shown in Figure 1. Four hundred twenty-three patients were found not eligible. The remaining 1,367 eligible patients were invited to participate, of whom 240 declined participation. Moreover, 85 patients had missing data on SF12. Hence 1,042 patients were included in the present study 2–36 (median 16) months after the index event.

### 2.2. Ethics

All participants gave signed informed consent before participation. The study was approved by The Regional Committee of Ethics in Medical Research (2013/1885).

### 2.3. Measures

Demographic and clinical data collected from the hospital records at the time of study inclusion were: Age, gender, coronary index diagnosis, ≥1 coronary event prior to the index event, participation in cardiac rehabilitation, other somatic comorbidities (i.e., stroke/transitory ischemic attack, peripheral artery disease, chronic kidney disease, heart failure, chronic obstructive pulmonary disease, inflammatory disease) and a diagnosis of diabetes.

A comprehensive self-report questionnaire comprised assessments of: living alone (yes/no), current smoking (defined as daily smokers at time of study inclusion), physical activity (defined as physical activity less than once a week) (Kurtze et al., 2008), eating fish (low intake was defined as less than three times a week), risk of obstructive sleep apnea; Berlin Questionnaire; (Munkhaugen et al., 2016), and taking sleep medication in the previous week (yes/no).

Health-related quality of life was assessed using the SF12, which is a 12-item questionnaire yielding two subscales: the MCS and the PCS (Ware et al., 1996). The MCS comprises items of vitality, social functioning, role-emotional and mental health. The PCS consists of items related to general physical functioning, role-physical, bodily pain, and general health (Ware et al., 1996). Scores of the MCS and PCS were calculated according to a standard scoring algorithm (Ware et al., 1995). SF12 has been validated in CHD patients (Mols et al., 2009) and Norwegian normative data exist (Gandek et al., 1998). In the NORCOR study, the 4-week test–retest reliability was 0.77 for PCS and 0.89 for MCS (Peersen et al., 2017).

Symptoms of anxiety and depression were assessed with the Hospital Anxiety and Depression Scale (HADS), which consist of two seven-item subscales (Zigmond and Snaith, 1983). Items are answered on four-point Likert scales ranging from 0 to 3. One subscale assesses anxiety symptoms (HADS-A) and the other subscale assesses depressive symptoms (HADS-D). A score of ≥8 on either of these two subscales was used as a cut-off value for clinically significant symptoms of anxiety or depression (Bjelland et al., 2002). The Norwegian version of the HADS has satisfactory psychometric properties, including in CHD populations (Bjelland et al., 2002). In the NORCOR study, the 4-week test–retest reliability was 0.92 for HADS-A and 0.94 for HADS-D (Peersen et al., 2017). Cronbach’s alphas were 0.83 for HADS-A, and 0.77 for HADS-D in the present study.

The type D Personality Scale (DS14) consists of two seven-item subscales assessing negative affectivity (NA) and social inhibition (SI) (Denollet, 2005). Items are answered on 5-point Likert scales ranging from 0 to 4. Type D personality is defined as having a score ≥10 on both the NA and SI subscales. The Norwegian version has been validated in CHD patients, with a test–retest reliability of 0.90 (SI subscale) and 0.91 (NA subscale) [over a 1-month period (Peersen et al., 2017)]. Cronbach’s alphas were 0.87 for the NA subscale and 0.83 for the SI subscale in the present study.

The Bergen Insomnia Scale (BIS) is a six-item questionnaire based on the criteria for the clinical diagnosis of insomnia described in the Diagnostic and Statistical manual, 4th version (Pallesen et al., 2008). The first four items enquire about difficulties with sleep initiation, maintenance of sleep, awakenings in the morning, and non-restorative sleep. A 30-min cut-off value is used for items one to three. Items five and six assess daytime impairment and dissatisfaction with sleep. Items are scored as number of days per week (0–7), yielding a continuous sum score from 0 to 42 (BIS sum score) increasing with symptom severity. The BIS questionnaire may also be used as a diagnostic tool (insomnia vs. no insomnia). Three days or more on items one, two, three or four combined with 3 days or more on items five or six indicate a diagnosis of insomnia. These were categorized as having “insomnia” whereas those who did not fulfill these criteria were categorized as insomnia negative (insomnia yes/no). The BIS has normative Norwegian data for comparison and adequate psychometric properties (Pallesen et al., 2008). In the NORCOR study, the 4-week test–retest reliability of the BIS was 0.92 (Peersen et al., 2017), and the Cronbach’s alpha was 0.88.

The standardized clinical examination included: Assessment of systolic and diastolic blood pressures using a validated digital sphygmomanometer (Welch Allyn WA Connex ProBP 3400), height (nearest 0.5 cm) and weight (nearest 0.5 kg), waist circumference (nearest 0.5 cm) as well as blood sampling including high-sensitivity C-reactive protein and low-density lipoprotein cholesterol (both analyzed with Architect ci16200, Abbott Laboratories, Abbott Park, IL, USA).

### 2.4. Statistics

Frequencies (%) and means with standard deviations (SD) were used for descriptive statistics, with chi-square tests to compare frequencies and independent samples *t*-test to test group differences.

To identify the subset of predictors in which all were significant when multi-variably controlled, a two-step elimination procedure was used to fit a linear regression model. Most variables presented in Table 1 were included in the first step. However, we did not include sleep medication in the past week as this may mediate the possible insomnia and QoL association. Nor did we include BIS, HADS-A, and HADS-D sum scores as the dichotomous variables (insomnia present, HADS-A or HADS-D ≥ 8 and Type-D personality present) were considered to be more clinically valid and could be easily applied and interpreted. Two supplementary tables including crude estimates for all included predictor variables for MCS and PCS are included in the Supplementary Tables 3, 4. The *p*-value was set at <0.10 for crude estimates to be included in the multi-adjusted model (second step). Age and sex were forced into final models (second step) after the elimination procedure had been completed (regardless of their level of significance), on the assumption that these variables would be putative confounders. To investigate the sensitivity of the enter model we also performed backward and forward stepwise regressions. We also calculated internal consistency for each questionnaire with standardized Cronbach’s alpha. A *p*-value < 0.05 was considered statistically significant in all analyses. The statistical analysis was performed using SPSS version 28.

**TABLE 1:** Characteristics.

	Total (*N* = 1042)
**Demographic factors**	
Age, mean (SD)	61 (10)
Female gender, *n* (%)	211 (20)
Months between index event and follow-up, mean (SD)	17 (11)
Low education (≤ 12 years), *n* (%)	719 (70)
Living alone, *n* (%)	184 (19)
Clinical factors	
Coronary index diagnosis	
Acute myocardial infarction, *n* (%)	825 (79)
Stable or unstable angina, *n* (%)	217 (21)
More than 1 coronary event prior to the index event, *n* (%)	312 (30)
Participation in cardiac rehabilitation, *n* (%)	528 (51)
**Comorbidity**	
Stroke/TIA, *n* (%)	69 (7)
Peripheral artery disease, *n* (%)	86 (8)
Chronic kidney disease, *n* (%)	132 (14)
Heart failure, *n* (%)	135 (13)
COPD, *n* (%)	93 (9)
Inflammatory disease, *n* (%)	79 (8)
**Coronary risk factors at interview**	
CRP ≥ 2 mg/L, *n* (%)*	395 (40)
Low density lipoprotein cholesterol >1.8 mmol/L, *n* (%)	579 (57)
Current smoking (“Røyking ved inklusjon”), *n* (%)	208 (21)
Diabetes, *n* (%)	175 (17)
Physical activity <1 times per week, *n* (%)	173 (17)
Systolic blood pressure, mmHg mean (SD)	138 (19)
Waist circumference obesity, *n* (%)	550 (59)
Eating fish <3 times/weeks, *n* (%)	484 (46)
OSA risk (yes/no), *n* (%)	406 (46)
Sleep medication past week *n* (%)	156 (15)
Psychosocial factors	
SF-12 MCS mean (SD)	47.1 (6)
SF-12 PCS mean (SD)	38.5 (5)
HADS-A sum score, mean (SD)	4.7 (4)
HADS-D sum score, mean (SD)	3.8 (3)
HADS-A ≥ 8, *n* (%)	211 (21)
HADS-D ≥ 8, *n* (%)	147 (14)
Type D personality, *n* (%)	185 (18)
Bergen insomnia scale sum, mean (SD)	13.8 (11)

*Excluded cases >15 mg/L.

COPD, chronic obstructive pulmonary disease; CRP, C-reactive protein; HADS-A, hospital anxiety and depression rating scale- anxiety subscale; HADS-D, hospital anxiety and depression rating scale- depression subscale; OSA, obstructive sleep apnea; SD, standard deviation; SF-12 MCS, the short form health survey mental component summary; SF-12 PCS, the short form health survey physical component summary; Type D, distressed type; TIA, transient ischemic attack.

## 3. Results

Baseline demographic, clinical and psychosocial characteristics in the study population are shown in Table 1. Mean age was 61 (SD 10) years, and 20% were females. Myocardial infarction was the index event in 806 patients (79%), while 211 patients (21%) had stable or unstable angina. Mean scores for SF12 subscales were 47 (SD 6) for MCS and 39 (SD 5) for PCS.

The results from crude and multi-adjusted linear regression analyses are presented in Table 2 for MCS and in Table 3 for PCS. The independent multi-adjusted covariates associated with reduced MCS were the presence of chronic kidney disease, insomnia, significant symptoms of depression and the presence of type D personality. Increased age at index event and longer duration between the index event and follow-up were associated with increased MCS. For reduced PCS, the independent multi-adjusted covariates were increased age, longer duration between the index event and follow-up, the presence of chronic obstructive pulmonary disease, and physical inactivity. The adjusted *R*^2^ was 0.18 for the MCS model and 0.05 for the PCS model.

**TABLE 2:** Mental component score regressed on study factors by linear regression analysis.

	Crude estimate			Multi-adjusted estimate^a^ (Adj. *R*^2^: 0.179)		
**Study factors**	**b (Standard error)**	**Standardized β**	***p*-value**	**b (Standard error)**	**Standardized β**	***p*-value**
Age at index event per year	0.12 (0.02)	0.20	<0.001	0.11 (0.02)	0.18	<0.001
Female gender	0.91 (0.46)	0.06	0.047	0.26 (0.50)	0.02	0.599
Months between index event and follow-up	0.03 (0.02)	0.06	0.051	0.06 (0.02)	0.11	0.002
Cardiac rehabilitation	0.66 (0.37)	0.06	0.074	-0.13 (0.40)	-0.01	0.756
Peripheral artery disease	-1.26 (0.67)	-0.06	0.060	-0.45 (0.77)	-0.02	0.559
Chronic kidney disease	-0.95 (0.55)	-0.06	0.086	-2.02 (0.63)	-0.11	0.001
Current smoking	-1.02 (0.46)	-0.07	0.027	0.01 (0.50)	0.00	0.978
Diabetes	-1.22 (0.49)	-0.08	0.013	-0.35 (0.55)	-0.02	0.531
SBP	0.02 (0.01)	0.08	0.020	0.00 (0.01)	0.00	0.918
Fish <3 times/weeks	-0.73 (0.37)	-0.06	0.047	-0.14 (0.39)	-0.01	0.724
OSA risk (yes/no)	-1.13 (0.39)	-0.10	0.004	0.06 (0.41)	0.01	0.883
HADS-A ≥ 8	-0.4.23 (0.44)	-0.29	<0.001	-0.56 (0.57)	-0.04	0.328
HADS-D ≥ 8	-5.30 (0.50)	-0.32	<0.001	-2.44 (0.64)	-0.15	<0.001
Type D personality	-4.97 (0.46)	-0.32	<0.001	-2.85 (0.57)	-0.19	<0.001
Insomnia	-3.14 (0.36)	-0.26	<0.001	-1.45 (0.43)	-0.13	<0.001

Study factors with *p* ≤ 0.1 in crude analyses are presented in the table. Unstandardized (b) and standardized (β) regression coefficients.

^a^Adjusted for all variables with *p* ≤ 0.1 retained in ENTER linear regression analysis with age and sex forced into the final model.

HADS-A, hospital anxiety and depression rating scale- anxiety subscale; HADS-D, hospital anxiety and depression rating scale- depression subscale; OSA, obstructive sleep apnea; SBP, systolic blood pressure; SD, standard deviation; Type D personality, distressed type personality.

**TABLE 3:** Physical component score regressed on study factors by linear regression analysis.

	Crude estimate			Multi-adjusted estimate^a^ (Adj. *R*^2^: 0.048)		
**Study factors**	**b (Standard error)**	**Standardized β**	***p*-value**	**b (Standard error)**	**Standardized β**	***p*-value**
Age at index event per year	-0.05 (0.02)	-0.09	0.003	-0.04 (0.02)	-0.08	0.016
Female gender	0.16 (0.37)	0.01	0.662	-0.22 (0.38)	-0.02	0.567
Low education (≤12 years)	-0.68 (0.32)	-0.07	0.036	-0.42 (0.33)	-0.04	0.208
Months between index event and follow-up	-0.04 (0.01)	-0.09	0.005	-0.04 (0.01)	-0.09	0.006
Stroke/TIA	-1.47 (0.59)	-0.08	0.013	-0.83 (0.61)	-0.04	0.173
Heart failure	-0.89 (0.44)	-0.06	0.042	-0.85 (0.45)	-0.06	0.059
COPD	-1.72 (0.52)	-0.10	<0.001	-1.24 (0.54)	-0.07	0.022
Current smoking	-0.90 (0.37)	-0.08	0.015	-0.48 (0.38)	-0.04	0.211
Physical inactivity	-2.01 (0.39)	-0.16	<0.001	-1.72 (0.41)	-0.14	<0.001

Study factors with p ≤ 0.1 in crude analyses are presented in the table. Unstandardized (b) and standardized (β) regression coefficients.

^a^Adjusted for all variables with p ≤ 0.1 retained in ENTER linear regression analysis with age and sex forced into the final model.

SD, standard deviation; TIA, transient ischemic attack; COPD, chronic obstructive pulmonary disease.

Sensitivity analyses in terms of stepwise backward and forward regressions (for MCS and PCS) hardly changed the estimates in the multi-adjusted models (data not shown), and the same independent multi-adjusted covariates remained significant.

Compared to SF12 responders, SF12 non-responders were older at the index event (mean years 64 vs. 61), were more often females (31 vs. 20%), reported lower education (78 vs. 70%), and reported higher prevalence rates of psychosocial variables (HADS-A, HADS-D, Type-D personality and insomnia) (Supplementary Table 1). Low to moderate correlations (0.00–0.46) were found between HRQoL (MCS and PCS) and psychological variables (HADS-A, HADS-D, Type-D, and insomnia) (Supplementary Table 2).

## 4. Discussion

In this cross-sectional study among CHD outpatients, we identified different determinants associated with the mental and physical component subscales of HRQoL. Type D personality, significant symptoms of depression, and insomnia were negatively associated with MCS in multi-adjusted analyses. Moreover, the presence of chronic kidney disease was also associated with reduced MCS, while increasing age and longer duration since index event were associated with increased MCS. Increasing age and longer duration since the index CHD event, the presence of chronic obstructive pulmonary disease, and low physical activity were all negatively associated with PCS. In total, the clinical and psychological variables explained 18% of the variance in scores of the mental component of HRQoL whereas these variables only explained 5% of the variance in scores of the physical component.

### 4.1. Study factors associated with MCS and PCS

Interestingly, type D personality, depression, and insomnia all remained significant in the final multi-adjusted model, which may indicate that all three factors are particularly independently important for the mental health component of HRQoL. Type D personality showed the strongest association with MCS in the multi-adjusted model, which supports the importance of type D personality as a key factor in reduced quality of life in CHD patients (Jo et al., 2019). Furthermore, our results of increased HADS-D being significantly correlated with reduced MCS scores are in line with those of previous studies (Muhammad et al., 2014).

We identified an association between the presence of insomnia diagnosis and reduced MCS, which is a novel finding. Two previous studies in CHD patients have reported that lower sleep quality, assessed with the Pittsburgh Sleep Quality Index (PSQI) (Buysse et al., 1989), was associated with lower quality of life as assessed by the EuroQol Questionnaire (Mei et al., 2021) and the Quality of Life after Myocardial Infarction Questionnaire (Shen et al., 2022). However, PSQI assesses sleep quality, which differs from insomnia diagnosis as assessed by the BIS. Moreover, different HRQoL questionnaires may assess different features, e.g., scores on the five dimensions in the EuroQol Questionnaire correlated low with the scores on the MCS and PCS of SF12 in CHD patients (De Smedt et al., 2014).

Surprisingly, the crude association between anxiety and the MCS score was no longer significant in multi-adjusted analyses. This contrasts the result of a previous study, which reported HADS-A scores to be associated with reduced MCS scores in CHD patients (Muhammad et al., 2014), even though that study reported lower mean scores of HADS-A and a lower prevalence rate of significant symptoms of anxiety (3% with HADS-A ≥ 8) together with a higher level of MCS compared to our study (Muhammad et al., 2014). Caseness of HADS-A and HADS-D have also been associated with reduced general HRQoL (SF36) in another study (Mayer et al., 2020). However, none of the previous studies adjusted for Type D personality and insomnia. Hence, we cannot confidently exclude anxiety as an important factor for mental HRQoL.

Despite a comprehensive set of study factors, our final model, which included age, time since index event and chronic kidney disease, in addition to the above-mentioned psychosocial variables, only explained 18% of the variance in the MCS scores. It is unknown which other potential factors could explain the remaining variance in MCS. In comparison, the study by Muhammad et al. (2014) found that HADS-A, HADS-D and age explained 33% of the variance in MCS scores in CHD patients. Furthermore, chronic kidney disease was associated with reduced MCS in our study, which is the first to report such a result among CHD patients. However, a similar finding has been reported in a in pre-dialytic chronic kidney disease patients (Alhaji et al., 2018) in which an association between chronic kidney disease and reduced PCS was also reported.

We identified physical inactivity, chronic obstructive pulmonary disease, increasing age and time since index event, as being significantly associated with PCS. Our model final modelonly explained 5% of the variance in PCS. This is surprisingly low with the comprehensive set of variables included in our study. Of particular interest, HADS-D was not associated with PCS. In contrast, Muhammad et al. (2014) found education and HADS-D to be associated with PCS in a multi-adjusted model which explained 22% of the variance in PCS.

Other studies on SF12 and SF36 in CHD patients have reported results both in line with, but also contradicting, our results (McBurney et al., 2002; Muhammad et al., 2014; Palacios et al., 2016; Mayer et al., 2020). Increasing age was associated positively with MCS and negatively with PCS in our study, in line with what was found in population-based studies (Gandek et al., 1998; Matsui et al., 2021). Mean MCS in our CHD population is lower than the normative data whereas the PCS scores are larger (Gandek et al., 1998). Our analysis yielded lower mean MCS (47 vs. 58) and mean PCS (39 vs. 48) compared with results from a study in CHD outpatients from Singapore (Muhammad et al., 2014). Furthermore, there is a clear discrepancy between the variables that were associated with MCS and PCS in our study, even though mental and physical quality of life most likely are closely correlated. However, the scoring algorithm of SF12 specifically has been designed to minimize this correlation (Ware et al., 1994).

### 4.2. Secondary prevention of CHD and HRQoL

Secondary prevention of CHD should focus on increasing HRQoL. Our study identified a novel finding of insomnia together with type D personality and depression being important to the mental aspects of HRQoL in CHD outpatients. This raises the question as to how many of these variables would optimally need to be targeted to improve the mental aspects of HRQoL? A recent Cochrane review reported an effect of cardiac rehabilitation on PCS and MCS, however, only 9 out of 47 studies incorporated psychological interventions and the authors questioned the clinical significance. Psychosocial interventions alone (or in addition to exercise) may improve HRQoL but have not been sufficiently tested in CHD patients (Dibben et al., 2021). Thus, at this stage, it is unclear which are the main factors to target in psychosocial intervention to improve HRQoL in these patients. Our study results indicate that managing type D personality, depression, and insomnia may all be important for the mental aspects of HRQoL in CHD patients. A recent Cochrane review found cognitive-behavioral therapy (CBT) for depression in CHD patients to decrease depression symptoms and improve short (<1 month post-treatment) and long term (>6 months post-treatment) MCS scores with moderate effect sizes (Tully et al., 2021). In the general population, CBT for chronic insomnia and medication (<4 weeks) for short-term insomnia are recommended (Riemann et al., 2017). A recent meta-analysis showed a moderate effect of CBT for chronic insomnia on quality of life, however, the effect in CHD patients were not reported (Alimoradi et al., 2022). Unfortunately, there is no recommended intervention for type D personality (ref). However, for depression in type D patients, psychotherapy has been recommended (Kupper and Denollet, 2018). Thus, to date, CBT seems to be the best documented treatment for depression and insomnia that were associated with reduced MCS in our study.

We found both depression and insomnia as being important and psychotherapy for depression has been reported to reduce insomnia symptoms (Mason et al., 2022) and vice versa (Cunningham and Shapiro, 2018; Mirchandaney et al., 2022). This leads to an intriguing possibility of treating several comorbid psychological conditions simultaneously. Metacognitive therapy has been reported as being effective in reducing symptoms of both anxiety and depression in CHD patients (Wells et al., 2021) and the attention training technique (one component of metacognitive therapy) has recently been described as feasible in a pilot study in CHD patients with some indication of both reducing symptoms of anxiety and depression as well as insomnia and the negative affectivity trait of Type D personality (Dammen et al., 2022). Such treatments should be tested in future randomized controlled trials including the assessments of HRQoL.

In addition to manage the mental conditions, more optimal management of somatic comorbidities with an emphasis on chronic kidney disease and chronic obstructive pulmonary disease may enhance the HRQoL as the presence of these diseases was associated with reduced MCS and PCS, respectively. Targeting physical inactivity may most likely improve HRQoL (Dibben et al., 2021) and should be addressed in cardiac out-patients.

### 4.3. Limitations

There are several limitations in this study. First, the cross-sectional design limits possibilities to attribute causality. Thus, the long-term effects on HRQoL if targeting the psychosocial variables associated with reduced HRQoL remains to be studied. Future prospective cohort studies and randomized controlled trials may address such questions. Second, confounding factors can never be excluded in observational studies. In our study the lack of objective assessment of obstructive sleep apnea (e.g., polysomnography) is most obvious, even though we included a screening questionnaire for obstructive sleep apnea (i.e., Berlin Questionnaire) (Netzer et al., 1999). Third, the psychosocial factors were based on self-report questionnaires which are easy to apply in clinical settings, but should not be interpreted as results from diagnostic interviews. Fourth, skewness of gender in the sample (80% men) limits the generalizability to women, because gender-specific predictors of HRQoL have been reported (Pettersen et al., 2008).

## 5. Conclusion

In our study, type D personality, depressive symptoms, and insomnia were the strongest predictors of the MCS, but not the PCS. Consequently, assessing and managing these psychological factors among CHD outpatients may thus improve their mental HRQoL and is therefore of clinical importance. Chronic obstructive pulmonary disease and low physical activity were modifiable predictors negatively associated with physical component. However, our model only explained 5% of the variance of PCS.

## Data availability statement

The datasets presented in the current manuscript are not available. We are not allowed to share original study data publicly according to Norwegian legislation, the Norwegian Data Protection Authority, and the Committee of Ethics. However, the essential generated data are available on request. Requests to access the datasets should be directed to LF, larsafr@uio.no.

## Ethics statement

The studies involving human participants were reviewed and approved by the Regional Committee of Ethics in Medical Research (2013/1885). The patients/participants provided their written informed consent to participate in this study.

## Author contributions

TD, JM, CP, and LF developed the idea and design and contributed to interpretation of results. JM and ES conducted the data collection. LF and TM conducted the statistical analyses. LF drafted the first version of the manuscript. All authors contributed to the final version of the manuscript.

## References

[B1] Alhaji M. Tan J. Hamid M. Timbuak J. Naing L. Tuah N. (2018). Determinants of quality of life as measured with variants of SF-36 in patients with predialysis chronic kidney disease. *Saudi. Med. J.* 39 653–661. 10.15537/smj.2018.7.21352 29968886 PMC6146254

[B2] Alimoradi Z. Jafari E. Broström A. Ohayon M. Lin C. Griffiths M. (2022). Effects of cognitive behavioral therapy for insomnia (CBT-I) on quality of life: a systematic review and meta-analysis. *Sleep Med. Rev.* 64:101646. 10.1016/j.smrv.2022.101646 35653951

[B3] American Psychiatric Association (2000). *Diagnostic and Statistical Manual of Mental Disorders DSM-IV-TR*, 4th Edn. Washington, DC: American Psychiatric Association.

[B4] Bjelland I. Dahl A. Haug T. Neckelmann D. (2002). The validity of the hospital anxiety and depression scale. An updated literature review. *J. Psychosom. Res.* 52 69–77. 11832252 10.1016/s0022-3999(01)00296-3

[B5] Buysse D. Reynolds C. III Monk T. Berman S. Kupfer D. (1989). The pittsburgh sleep quality index: a new instrument for psychiatric practice and research. *Psychiatry Res.* 28 193–213. 10.1016/0165-1781(89)90047-4 2748771

[B6] Christensen A. Juel K. Ekholm O. Thrysøe L. Thorup C. Borregaard B. (2020). Significantly increased risk of all-cause mortality among cardiac patients feeling lonely. *Heart* 106 140–146. 31685646 10.1136/heartjnl-2019-315460

[B7] Coryell V. Ziegelstein R. Hirt K. Quain A. Marine J. Smith M. (2013). Clinical correlates of insomnia in patients with acute coronary syndrome. *Int. Heart J.* 54 258–265. 24097213 10.1536/ihj.54.258

[B8] Cunningham J. Shapiro C. (2018). Cognitive behavioural therapy for insomnia (CBT-I) to treat depression: a systematic review. *J. Psychosom. Res.* 106 1–12. 29455893 10.1016/j.jpsychores.2017.12.012

[B9] Da Costa D. Allman A. Libman E. Desormeau P. Lowensteyn I. Grover S. (2017). Prevalence and determinants of insomnia after a myocardial infarction. *Psychosomatics* 58 132–140. 28104338 10.1016/j.psym.2016.11.002

[B10] Dammen T. Tunheim K. Munkhaugen J. Papageorgiou C. (2022). The attention training technique reduces anxiety and depression in patients with coronary heart disease: a pilot feasibility study. *Front. Psychol.* 13:948081. 10.3389/fpsyg.2022.948081 35967654 PMC9363691

[B11] De Smedt D. Clays E. Annemans L. De Bacquer D. (2014). EQ-5D versus SF-12 in coronary patients: are they interchangeable? *Value Health* 17 84–89. 10.1016/j.jval.2013.10.010 24438721

[B12] Denollet J. (2000). Type D personality. A potential risk factor refined. *J. Psychosom. Res.* 49 255–266. 10.1016/s0022-3999(00)00177-x 11119782

[B13] Denollet J. (2005). DS14: standard assessment of negative affectivity, social inhibition, and Type D personality. *Psychosom. Med.* 67 89–97. 15673629 10.1097/01.psy.0000149256.81953.49

[B14] Dibben G. Faulkner J. Oldridge N. Rees K. Thompson D. Zwisler A. (2021). Exercise-based cardiac rehabilitation for coronary heart disease. *Cochrane Database Syst. Rev.* 11:Cd001800. 10.1002/14651858.CD001800.pub4 PMC8571912 34741536

[B15] Franquelo-Morales P. Sánchez-López M. Notario-Pacheco B. Miota-Ibarra J. Lahoz-García N. Gómez-Marcos M. Á (2018). Association between health-related quality of life, obesity, fitness, and sleep quality in young adults: the cuenca adult study. *Behav. Sleep Med.* 16 347–355. 10.1080/15402002.2016.1228638 27754696

[B16] Frøjd L. Dammen T. Munkhaugen J. Weedon-Fekjær H. Nordhus I. Papageorgiou C. (2022). Insomnia as a predictor of recurrent cardiovascular events in patients with coronary heart disease. *SLEEP Advances* 3:zac007. 10.1093/sleepadvances/zpac007 PMC10104412 37193392

[B17] Frøjd L. Munkhaugen J. Moum T. Sverre E. Nordhus I. Papageorgiou C. (2021). Insomnia in patients with coronary heart disease: prevalence and correlates. *J. Clin. Sleep Med.* 17 931–938. 33399066 10.5664/jcsm.9082 PMC8320477

[B18] Gandek B. Ware J. Aaronson N. Apolone G. Bjorner J. Brazier J. (1998). Cross-validation of item selection and scoring for the SF-12 Health Survey in nine countries: results from the IQOLA project. International quality of life assessment. *J. Clin. Epidemiol.* 51 1171–1178. 10.1016/s0895-4356(98)00109-7 9817135

[B19] Javaheri S. Redline S. (2017). Insomnia and risk of cardiovascular disease. *Chest* 152 435–444. 28153671 10.1016/j.chest.2017.01.026 PMC5577359

[B20] Jo E. Kim S. Kim H. (2019). Predictive model for quality of life in patients with recurrent coronary artery disease. *Eur. J. Cardiovasc. Nurs.* 18 501–511. 31044610 10.1177/1474515119847544

[B21] Kupper N. Denollet J. (2018). Type D personality as a risk factor in coronary heart disease: a review of current evidence. *Curr. Cardiol. Rep.* 20:104. 10.1007/s11886-018-1048-x 30209683 PMC6153564

[B22] Kurtze N. Rangul V. Hustvedt B. Flanders W. (2008). Reliability and validity of self-reported physical activity in the Nord-Trøndelag Health Study: HUNT 1. *Scand. J. Public Health* 36 52–61. 10.1177/1403494807085373 18426785

[B23] Le Grande M. Jackson A. Murphy B. Thomason N. (2016). Relationship between sleep disturbance, depression and anxiety in the 12 months following a cardiac event. *Psychol. Health Med.* 21 52–59. 25958936 10.1080/13548506.2015.1040032

[B24] Mason E. Grierson A. Sie A. Sharrock M. Li I. Chen A. (2022). Co-occurring insomnia and anxiety: a randomized controlled trial of internet cognitive behavioral therapy for insomnia versus internet cognitive behavioral therapy for anxiety. *Sleep* 46:zsac205. 10.1093/sleep/zsac205 36041459

[B25] Matsui K. Yoshiike T. Nagao K. Utsumi T. Tsuru A. Otsuki R. (2021). Association of subjective quality and quantity of sleep with quality of life among a general population. *Int. J. Environ. Res. Public Health* 18:12835. 10.3390/ijerph182312835 34886562 PMC8657737

[B26] Mayer O. Jr. Bruthans J. Seidlerová J. Karnosová P. Mateřánková M. Gelžinský J. (2020). Mood disorders impaired quality of life but not the mortality or morbidity risk in stable coronary heart disease patients. *Acta Cardiol.* 75 667–675. 31442189 10.1080/00015385.2019.1653568

[B27] McBurney C. Eagle K. Kline-Rogers E. Cooper J. Mani O. Smith D. (2002). Health-related quality of life in patients 7 months after a myocardial infarction: factors affecting the Short Form-12. *Pharmacotherapy* 22 1616–1622. 10.1592/phco.22.17.1616.34121 12495171

[B28] Mei Y. Wu H. Zhang H. Hou J. Zhang Z. Liao W. (2021). Health-related quality of life and its related factors in coronary heart disease patients: results from the Henan rural cohort study. *Sci. Rep.* 11:5011. 10.1038/s41598-021-84554-6 33658589 PMC7930256

[B29] Mensah G. Wei G. Sorlie P. Fine L. Rosenberg Y. Kaufmann P. (2017). Decline in cardiovascular mortality. *Circ. Res.* 120 366–380. 28104770 10.1161/CIRCRESAHA.116.309115 PMC5268076

[B30] Mirchandaney R. Barete R. Asarnow L. (2022). Moderators of cognitive behavioral treatment for insomnia on depression and anxiety outcomes. *Curr. Psychiatry Rep.* 24 121–128. 35061137 10.1007/s11920-022-01326-3 PMC8948126

[B31] Mols F. Pelle A. Kupper N. (2009). Normative data of the SF-12 health survey with validation using postmyocardial infarction patients in the Dutch population. *Qual. Life Res.* 18 403–414. 10.1007/s11136-009-9455-5 19242822

[B32] Muhammad I. He H. Koh K. Thompson D. Kowitlawakul Y. Wang W. (2014). Health-related quality of life and its predictors among outpatients with coronary heart disease in Singapore. *Appl. Nurs. Res.* 27 175–180. 25052181 10.1016/j.apnr.2013.11.008

[B33] Munkhaugen J. Sverre E. Peersen K. Gjertsen E. Gullestad L. Moum T. (2016). The role of medical and psychosocial factors for unfavourable coronary risk factor control. *Scand. Cardiovasc. J.* 50 1–8. 26488672 10.3109/14017431.2015.1111408

[B34] Netzer N. Stoohs R. Netzer C. Clark K. Strohl K. (1999). Using the Berlin questionnaire to identify patients at risk for the sleep apnea syndrome. *Ann. Intern. Med.* 131 485–491. 10507956 10.7326/0003-4819-131-7-199910050-00002

[B35] Palacios J. Khondoker M. Achilla E. Tylee A. Hotopf M. (2016). A single, one-off measure of depression and anxiety predicts future symptoms, higher healthcare costs, and lower quality of life in coronary heart disease patients: analysis from a multi-wave, primary care cohort study. *PLoS One* 11:e0158163. 10.1371/journal.pone.0158163 27463115 PMC4963085

[B36] Palacios J. Khondoker M. Mann A. Tylee A. Hotopf M. (2018). Depression and anxiety symptom trajectories in coronary heart disease: associations with measures of disability and impact on 3-year health care costs. *J. Psychosom. Res.* 104 1–8. 10.1016/j.jpsychores.2017.10.015 29275777

[B37] Pallesen S. Bjorvatn B. Nordhus I. Sivertsen B. Hjørnevik M. Morin C. (2008). A new scale for measuring insomnia: the Bergen insomnia scale. *Percept. Mot. Skills* 107 691–706. 19235401 10.2466/pms.107.3.691-706

[B38] Peersen K. Munkhaugen J. Gullestad L. Dammen T. Moum T. Otterstad J. (2017). Reproducibility of an extensive self-report questionnaire used in secondary coronary prevention. *Scand. J. Public Health* 45 269–276. 10.1177/1403494816688375 28181463 PMC5405837

[B39] Pettersen K. Reikvam A. Rollag A. Stavem K. (2008). Understanding sex differences in health-related quality of life following myocardial infarction. *Int. J. Cardiol.* 130 449–456. 18221803 10.1016/j.ijcard.2007.10.016

[B40] Riemann D. Baglioni C. Bassetti C. Bjorvatn B. Dolenc Groselj L. Ellis J. (2017). European guideline for the diagnosis and treatment of insomnia. *J. Sleep Res.* 26 675–700. 28875581 10.1111/jsr.12594

[B41] Roth G. Mensah G. Johnson C. Addolorato G. Ammirati E. Baddour L. (2020). Global burden of cardiovascular diseases and risk factors, 1990–2019: update from the GBD 2019 study. *J. Am. Coll. Cardiol.* 76 2982–3021. 33309175 10.1016/j.jacc.2020.11.010 PMC7755038

[B42] Shen B. Tan J. Xu Y. Tay H. (2022). Poor sleep quality predicts decline in physical health functioning in patients with coronary heart disease and moderating role of social support. *Behav. Med.* 48 294–304. 10.1080/08964289.2021.1895050 33750280

[B43] Tang N. Fiecas M. Afolalu E. Wolke D. (2017). Changes in sleep duration, quality, and medication use are prospectively associated with health and well-being: analysis of the UK household longitudinal study. *Sleep* 40:zsw079. 10.1093/sleep/zsw079 28364423

[B44] Tully P. Ang S. Lee E. Bendig E. Bauereiß N. Bengel J. (2021). Psychological and pharmacological interventions for depression in patients with coronary artery disease. *Cochrane Database Syst. Rev.* 12:CD008012. 10.1002/14651858.CD008012.pub4 PMC8673695 34910821

[B45] Visseren F. Mach F. Smulders Y. Carballo D. Koskinas K. Bäck M. (2021). 2021 ESC guidelines on cardiovascular disease prevention in clinical practice: developed by the task Force for cardiovascular disease prevention in clinical practice with representatives of the European Society of Cardiology and 12 medical societies with the special contribution of the European Association of Preventive Cardiology (EAPC). *Eur. Heart J.* 42 3227–3337.34458905

[B46] Ware J. Jr. Gandek B. (1998). Overview of the SF-36 health survey and the international quality of life Assessment (IQOLA) Project. *J. Clin. Epidemiol.* 51 903–912. 10.1016/s0895-4356(98)00081-x 9817107

[B47] Ware J. Jr. Kosinski M. Keller S. D. (1996). A 12-item short-form health survey: construction of scales and preliminary tests of reliability and validity. *Med. Care* 34 220–233. 8628042 10.1097/00005650-199603000-00003

[B48] Ware J. Kosinski M. Keller S. (1994). *SF-36 Physical and Mental Summary Scales: a user‘s manual.* Boston, MA: The Health Institute.

[B49] Ware J. Kosinski M. Keller S. (1995). *SF-12: How to Score the SF-12 Physical and Mental Health Summary scales.* Boston, MA: The Health Institute.

[B50] Wells A. Reeves D. Capobianco L. Heal C. Davies L. Heagerty A. (2021). Improving the effectiveness of psychological interventions for depression and anxiety in cardiac rehabilitation: PATHWAY—a single-blind, parallel, randomized, controlled trial of group metacognitive therapy. *Circulation* 144 23–33. 10.1161/CIRCULATIONAHA.120.052428 34148379 PMC8247550

[B51] Westin L. Nilstun T. Carlsson R. Erhardt L. (2005). Patients with ischemic heart disease: quality of life predicts long-term mortality. *Scand. Cardiovasc. J.* 39 50–54. 16097414 10.1080/14017430410003903

[B52] Xie J. Wu E. Zheng Z. Sullivan P. Zhan L. Labarthe D. (2008). Patient-reported health status in coronary heart disease in the United States: age, sex, racial, and ethnic differences. *Circulation* 118 491–497. 10.1161/CIRCULATIONAHA.107.752006 18625894

[B53] Zigmond A. Snaith R. (1983). The hospital anxiety and depression scale. *Acta Psychiatr. Scand.* 67 361–370. 6880820 10.1111/j.1600-0447.1983.tb09716.x

