# Supplementary material for: Predictors of health-related quality of life in outpatients with coronary heart disease
Source: Front Psychol. 2023 Jun 9;14:1119093. doi: 10.3389/fpsyg.2023.1119093 (PMC10289018; doi:10.3389/fpsyg.2023.1119093)
Supplement: Supplementary file 1 [file Data_Sheet_1.docx]

| **Supplemental table S1.** Sociodemographic, clinical and psychosocial characteristics in coronary patients with missing data on SF12 and completed SF12. | | |
| --- | --- | --- |
|  | Missing data on SF12 (n=85) | *Completed SF12 (n=1042)* |
| ***Sociodemographic factors*** |  |  |
| Age, mean (SD) | 64 (9) | 61 (10) |
| Female gender, n (%) | 26 (31) | 211 (20) |
| Months between index event and follow-up, mean (SD) | 16 (10) | 17 (11) |
| ***Clinical factors*** |  |  |
| Chronic Kidney Disease, n (%) | 7 (9) | 132 (14) |
| COPD, n (%) | 6 (7) | 93 (9) |
| Physical activity < 1 times per week, n (%) | 24 (32) | 173 (17) |
| OSA risk (yes/no) , n (%) | 30 (53) | 406 (46) |
| ***Psychosocial factors*** |  |  |
| HADS-A ≥8, n (%) | 16 (26) | 211 (21) |
| HADS-D ≥8, n (%) | 12 (18) | 147 (14) |
| Type D personality, n (%) | 12 (19) | 185 (18) |
| Insomnia, n (%) | 31 (48) | 457 (45) |
| COPD, Chronic obstructive pulmonary disease  HADS-A, hospital anxiety and depression rating scale- anxiety subscale  HADS-D, hospital anxiety and depression rating scale- depression subscale  OSA, Obstructive sleep apnea  SD, standard deviation  SF-12 the short form health survey  Type D, distressed type personality | | |

| **Supplemental table S2.** Pearson correlations. | | | | | | |
| --- | --- | --- | --- | --- | --- | --- |
|  | Insomnia | HADS Anxiety | HADS Depression | Type D personlity | SF12-MCS | SF12-PCS |
| Insomnia | - | .34** | .23 ** | .23 ** | -.26** | -.00 |
| HADS-A |  | - | .39 ** | .46 ** | -.28 ** | .01 |
| HADS-D |  |  | - | .40 ** | -.31 ** | .03 |
| Type D |  |  |  | - | -.32 ** | .05 |
| SF12-MCS |  |  |  |  | - | -.41 ** |
| SF12-PCS |  |  |  |  |  | - |
| ** p<0.01  Type D, distressed type personality  HADS-A, hospital anxiety and depression rating scale- anxiety subscale  HADS-D, hospital anxiety and depression rating scale- depression subscale  SF12-MCS, Short Form 12 Mental Component Scale  SF12-PCS, Short Form 12 Physical Component Scale | | | | | | |

| **Table S3.** Mental Component Score regressed on study factors by linear regression analysis**.** | | | |
| --- | --- | --- | --- |
|  | Crude estimate | | |
| **Study factors** | b (standard error) | Standar-dized *β* | *p*-value |
| ***Demographic factors*** |  |  |  |
| Age at index event, per year | .12 (.02) | .20 | <0.001 |
| Female gender | .91 (.46) | .06 | .047 |
| Months between index event and follow-up | .03 (.02) | .06 | *.051* |
| Low education (≤ 12 years) | -.23 (.40) | -.02 | *.574* |
| Living alone | .45 (.48) | .03 | *.346* |
| ***Clinical factors*** |  |  |  |
| Coronary index diagnosis^2^ | .33 (.45) | .02 | *.471* |
| More than 1 coronary event prior to the index event ^3^ | -.20 (.40) | -.02 | *.615* |
| Cardiac rehabilitation ^4^ | .66 (.37) | .06 | *.074* |
| **Comorbidity** |  |  |  |
| Stroke/TIA | -.05 (.74) | -.00 | *.952* |
| Peripheral artery disease | -1.26 (.67) | -.06 | *.060* |
| Kidney failure | -.95 (.55) | -.06 | *.086* |
| Heart failure | -.80 (.55) | .05 | *.144* |
| COPD | .15 (.65) | .01 | *.814* |
| Inflammatory disease | -.49 (.70) | -.02 | *.481* |
| **Coronary risk factors at interview** |  |  |  |
| CRP ≥2 mg/L | -.59 (.39) | -.05 | *.125* |
| Low density lipoprotein cholesterol >1.8 mmol/L | .09 (.38) | .01 | *.815* |
| Current smoking | -1.02 (.46) | -.07 | *.027* |
| Diabetes | -1.22 (.49) | -.08 | *.013* |
| Physical activity < 1 times per week | -.69 (.50) | -.04 | *.163* |
| SBP | .02 (.01) | .08 | *.020* |
| Waist circumference obesity | -.42 (.39) | -.04 | *.276* |
|  |  |  |  |
| Fish < 3 times/wk | -.73 (.37) | -.06 | *0.047* |
| OSA risk (yes/no) | -1.13 (.39) | -.10 | *0.004* |
| ***Psychosocial factors*** |  |  |  |
| HADS-A ≥ 8 | -.4.23 (.44) | -.29 | *<0.001* |
| HADS-D ≥ 8 | -5.30 (.50) | -.32 | *<0.001* |
| Type D personality^13^ | -4.97 (.46) | -.32 | *<0.001* |
| Insomnia | -3.14 (-.36) | -.26 | *<0.001* |

| **Table S4.** Physical Component Score regressed on study factors by linear regression analysis. | | | |
| --- | --- | --- | --- |
|  | Crude estimate | | |
| **Study factors** | b (standard error) | Standar-dized *β* | *p*-value |
| ***Demographic factors*** |  |  |  |
| Age at index event, per year | -.05 (.02) | -.09 | .003 |
| Female gender | .16 (.37) | .01 | *.662* |
| Months between index event and follow-up | -.04 (.01) | -.09 | *.005* |
| Low education (≤ 12 years) | -.68 (.32) | -.07 | *.036* |
| Living alone | .45 (.39) | .04 | *.252* |
| ***Clinical factors*** |  |  |  |
| Coronary index diagnosis | .54 (.36) | .05 | *.141* |
| More than 1 coronary event prior to the index event | -.10 (.32) | -.01 | *.760* |
| Participation in cardiac rehabilitation | -.08 (.30) | -.01 | *.792* |
| **Comorbidity** |  |  |  |
| Stroke/TIA | -1.47 (.59) | -.08 | *.013* |
| Peripheral artery disease | .83 (.54) | .05 | *.123* |
| Chronic Kidney Disease | -.05 (.45) | -.00 | *.908* |
| Heart failure | -.89 (.44) | -.06 | *.042* |
| COPD | -1.72 (.52) | -.10 | *<0.001* |
| Inflammatory disease | -.41 (.56) | -.02 | *0.463* |
| **Coronary risk factors at interview** |  |  |  |
| CRP ≥2 mg/L | -.27 (.31) | -.03 | *.390* |
| Low density lipoprotein cholesterol >1.8 mmol/L | .30 (.30) | .03 | *.323* |
| Current smoking | -.90 (.37) | -.08 | *.015* |
| Diabetes | -.20 (.40) | -.02 | *.613* |
| Physical activity < 1 times per week | -2.01 (.39) | -.16 | *<0.001* |
| Systolic blood pressure | .00 (.01) | .02 | *.609* |
| Waist circumference obesity | -.51 (.32) | -.05 | *.108* |
| Eating fish < 3 times/wk | -.24 (.30) | -.03 | *.409* |
| OSA risk (yes/no) | .16 (.32) | .02 | *.632* |
| ***Psychosocial factors*** |  |  |  |
| HADS-A ≥ 8 | .22 (.37) | .02 | *.554* |
| HADS-D ≥ 8 | .49 (.42) | .04 | *.244* |
| Type D personality | .63 (.39) | .05 | *.103* |
| Insomnia | -.01 (.30) | .00 | *.980* |
